# Hierarchy in Structuring of Resource Selection: Understanding Elk Selection Across Space, Time, and Movement Strategies

**DOI:** 10.1002/ece3.71097

**Published:** 2025-03-05

**Authors:** Storm Crews, Nathaniel D. Rayl, Mathew W. Alldredge, Eric J. Bergman, Chuck R. Anderson, Eric H. VanNatta, Joseph D. Holbrook, Guillaume Bastille‐Rousseau

**Affiliations:** ^1^ Cooperative Wildlife Research Laboratory Southern Illinois University Carbondale Illinois USA; ^2^ School of Biological Sciences Southern Illinois University Carbondale Illinois USA; ^3^ Mammals Research Section Colorado Parks and Wildlife Grand Junction Colorado USA; ^4^ Mammals Research Section Colorado Parks and Wildlife Fort Collins Colorado USA; ^5^ Terrestrial Section Colorado Parks and Wildlife Steamboat Springs Colorado USA; ^6^ Haub School of Environment and Natural Resources University of Wyoming Laramie Wyoming USA; ^7^ Department of Zoology and Physiology University of Wyoming Laramie Wyoming USA

**Keywords:** consistency, GPS telemetry, habitat selection function, movement ecology, net squared displacement, normalized difference vegetation index (NDVI), partial migration, ungulate

## Abstract

Movement is a fundamental aspect of animal ecology that varies across space, time, and among individuals or groups within a population. Broad‐scale patterns of animal movement are often classified into different movement strategies, such as resident, nomadic, or migratory. While landscape‐level environmental patterns can predict the presence of different movement strategies in an area, elucidating how these patterns downscale to fine‐scale resource selection behaviors remains a challenge. Partially migratory systems, where both migrants and residents coexist, offer a unique opportunity to address these questions. Using tracking data from four Rocky Mountain elk (*Cervus canadensis)* herds situated primarily within Colorado, USA, we assessed between‐herd, seasonal, and within‐herd variation in resource selection behavior. We modeled fine‐scale seasonal resource selection and compared strategy‐specific behaviors using resource selection functions. Additionally, we used a consistency score to quantify the extent of differentiation in resource selection behavior across strategies, seasons, herds, and groups of covariates. We found variation in strategy frequency within each herd and in selection behavior, highlighting the complexity and context‐dependence of strategy‐specific selection. Despite herd‐specific differences, some consistent trends emerged, with elk avoiding human development and roads at fine scales while selecting areas with higher productivity during summer. Our consistency analysis identified where elk most diverged in their selection behavior, revealing the greatest differences among herds, followed by variation between seasons, and lastly between movement strategies. Elk exhibited more uniform responses to productivity, contrasting with greater differentiation in responses to anthropogenic‐related covariates. Overall, our study improves our understanding of elk behavior across space, time, and movement strategies and sheds light on the hierarchical influences of space and time in constraining behavior.

## Graphical abstract

We leveraged a unique dataset of over 500 elk from four herds living in very different environmental conditions, ranging from semi‐arid desert to high‐elevation mountains across the state of Colorado, USA. For each herd, we characterized broad movement strategies (whether individuals were migrant or resident) as well as fine‐scale resource selection during the winter (when residents and migrants overlapped in space) and summer (when residents and migrants did not overlap). Our work reveals nuances in elk behavior across space, herds, seasons, and movement strategies while also providing information on the hierarchy of those factors in constraining elk resource selection.

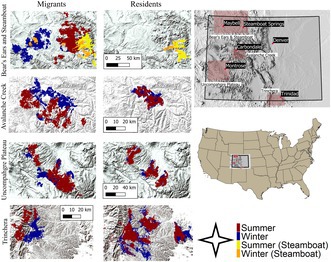

## 1 Introduction

Movement is a core component of animal ecology that can vary across space, time, and individuals or groups within a population. Movement strategies are broad‐scale movement behaviors exhibited by animals that should maximize fitness benefits and minimize costs in the context of the surrounding environment (Bastille‐Rousseau et al. 2015a; Nathan et al. 2008; Shaw 2020). These strategies encompass a spectrum of behaviors, commonly categorized as (1) resident, where animals maintain defined home ranges year‐round, (2) migrant, involving seasonal shifts between distinct and often discontinuous ranges, and (3) alternative, involving tactics such as nomadism or dispersal (Bunnefeld et al. 2011; Chapman et al. 2011). The existence and prevalence of these movement strategies are often driven by the spatiotemporal variation in abundance, variability, and predictability of resources in the broader environment (Bastille‐Rousseau et al. 2017; Mueller et al. 2011).

While clear predictions exist regarding how landscape‐level patterns should influence the presence of different movement strategies (Mueller et al. 2011), understanding how these broader environmental patterns translate into specific fine‐scale behaviors such as resource selection remains a challenge. Resource selection is a key concept in spatial ecology (McLoughlin et al. 2010). Patterns in resource selection contribute to our understanding of a species' habitat and potential risk‐foraging tradeoffs individuals balance (Brown 1999; Laundré 2010). Because broad movement strategies emerge from a succession of fine‐scale decisions regarding what to use, which also constrain what is ultimately available to an individual, the link between resource selection and movement strategies is complex. For example, given that migrants show non‐contiguous seasonal space use while residents remain in a defined area year‐round, differing resource availability is expected during allopatric periods where differences in selection might be driven by differences in availability, but not use (Hebblewhite and Merrill 2009). Even in systems where different strategies overlap within a given spatial and temporal context, however, sympatric individuals may not select for similar resources (Peterson et al. 2022). The extent of differentiation in selection across strategies can shed light on the type of interactions faced by animals (e.g., competition) and additional evolutionary drivers of their prevalence.

Partially migratory populations (populations with both migrants and residents) are uniquely suited to explore questions about how movement tactics influence habitat selection (Merrill et al. 2020). These systems generally have one season where both strategies overlap more closely in space and another season where each strategy occupies a distinct range. Many studies have shown how resources selected by each strategy during the allopatric period can differ (e.g., Hebblewhite and Merrill 2009; Middleton et al. 2013), yet comparing resource selection during the sympatric period is less common (Merrill et al. 2020). Comparing the behavior of migrants and residents during the sympatric period could be informative, especially when differences in selection are observed. Perhaps more limiting to our understanding, differentiation and similarity in behavior between movement strategies are most often focused on a single population. Evaluating the similarity in behavior of different movement strategies across varying temporal and spatial contexts, however, offers a broader comparative approach to contextualize the drivers and cost‐benefits of different strategies. More pragmatically, identifying resource selection between differing movement strategies may also aid the management of partially migratory populations by facilitating approaches that account for intrapopulation diversity in resource requirements if such diversity is significant.

Elk (*Cervus canadensis*) are ideally suited to study these questions given they often exhibit partial migration (Berg et al. 2019; Merrill et al. 2020). Studies have documented differences in exposure to risk (Hebblewhite and Merrill 2007; Middleton et al. 2013), forage access (Barker 2018), and calf survival (Berg et al. 2019) among movement tactics within a population. For example, in some systems, resident elk have shown greater affinity for human development, allowing them to avoid predation risk more successfully than their migratory counterparts, but at the cost of reduced access to quality forage (Hebblewhite and Merrill 2011). Other populations of resident elk also seem to leverage irrigated crops to offset the lower food quality available in their range during summer (Barker 2018). Given the plastic nature of elk movement tactics, the ratio of migrants and residents within herds may shift over time (Zuckerman et al. 2023), with pressures such as density‐dependence and predation risk influencing prevalence (Hebblewhite and Merrill 2011; Williams et al. 2023).

Here, we used tracking data from four elk herds mostly confined to Colorado, USA, and evaluated between‐herd, seasonal, and within‐herd variation in resource selection. First, we classified movement strategies of each elk‐year as either migratory or resident and assessed herd‐level strategy composition. We modeled fine‐scale seasonal resource selection of individuals to compare strategy‐specific resource selection. To provide a broader context for the structure of variation in behavior, we used an index of consistency in resource selection behavior to quantify the degree of differentiation in behavior between strategy, season, across herds, and across different groups of covariates (Bastille‐Rousseau and Wittemyer 2022). We predicted that resource selection would differ between movement strategies, with measurable differences in selection for anthropogenic, habitat, and productivity resources. We also predicted that the degree of differentiation would be highest among herds but that selection patterns would be similar between season and strategies. Overall, our study improves our understanding of elk behavior across herds, seasons, and movement strategies and elucidates the hierarchical constraints governing their resource selection.

## 2 Methods

### 2.1 Study Area

**FIGURE 1:**
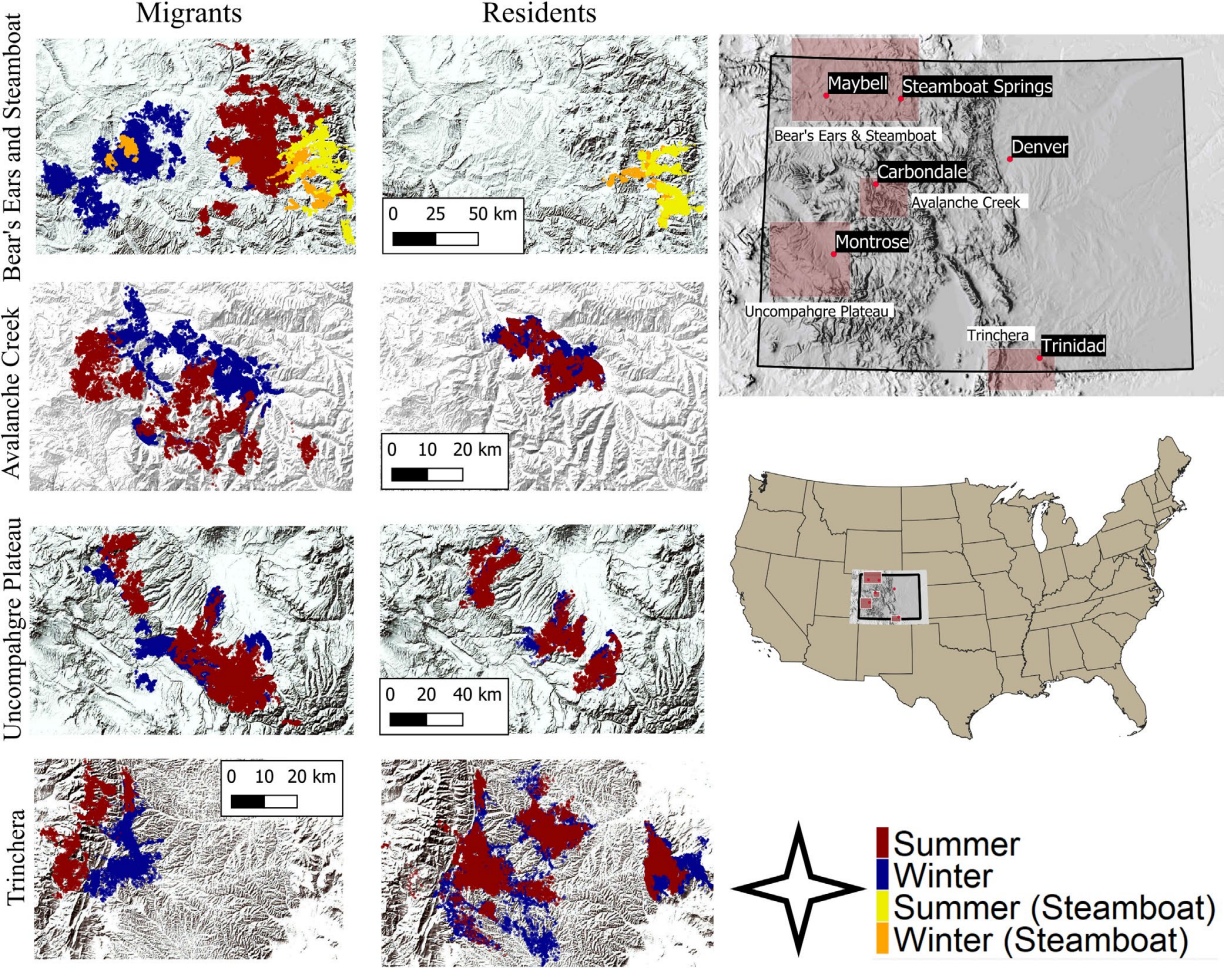
Locations of migratory and resident elk during winter (blue) and summer (red) 2017–2021 from the Avalanche Creek (DAU E‐15), Bear's Ears (DAU E‐2), Trinchera (DAU E‐33), and Uncompahgre Plateau (DAU E‐20) elk herds that wintered in Colorado, USA. Note that DAU E‐2 includes both the Bear's Ears and Steamboat analytical populations, with Steamboat winter and summer points shown in orange and yellow, respectively. The only residents observed in DAU E‐2 were Steamboat elk. Shading depicts hillshade of elevation in all maps.

We studied elk from the Avalanche Creek (Colorado Parks and Wildlife Data Analysis Unit [DAU] E‐15), Bear's Ears (DAU E‐2), Trinchera (DAU E‐33), and Uncompahgre Plateau (DAU E‐20) herds that wintered in Colorado, USA (Figure 1). We captured elk from the Bear's Ears herd on two distinct winter ranges. Although these elk occupied the same DAU, we considered them as separate analytical units due to the geographic separation of their winter ranges (approximately 110 km apart). Hereafter, we refer to elk caught on the western and eastern sections of DAU E‐2 as the “Bear's Ears” and “Steamboat” herds, respectively (Figure 1). Elk from the Bear's Ears herd occasionally crossed into Wyoming, and elk from the Trinchera herd often crossed into New Mexico. The range of all herds occurred within the Southern Rockies ecoregion. Additionally, the ranges of the Bear's Ears and Uncompahgre Plateau herds occurred within the Colorado Plateau and Wyoming Basin ecoregions (Environmental Protection Agency 2021). As observed in other elk populations, migratory elk in Colorado generally winter at low elevation and summer at higher elevation, while resident elk remain year‐round on the same range, or regularly commuted between two ranges. Population estimates (ca. 2021) for each herd are: Avalanche Creek (AC) ~4800, Bear's Ears (BE) ~18,400, Steamboat (SB) ~600–800, Trinchera (TR) ~14,000, and Uncompahgre Plateau (UP) ~12,500.

Elevations range from ~1500 m in low‐lying areas to > 4000 m at mountain peaks, with diverse vegetative communities largely determined by altitude (Peet 1978). At low elevations (< 2000 m) pinyon pine (*Pinyon edulis*)–Utah juniper ( *Juniperus osteosperma* ) forests, plains grasslands, sagebrush steppe, higher desert, desert basins, and river valleys are found. Moderate elevations (2000–3500 m) are characterized by mountain shrubs, sagebrush, aspen or mixed aspen/conifer forests, and spruce and fir communities. High elevations (> 3500 m) are characterized by alpine flora or talus slopes. The study area is a mosaic of public and private land ownership.

### 2.2 Animal Capture and Monitoring

We opportunistically captured yearling and adult female elk on winter range by helicopter net‐gunning or darting during January–March, 2017–2021. We radio‐collared elk with Global Positioning System (GPS) collars programmed to acquire a location every 1, 2, or 4 h (G2110E2, G5‐2D, Advanced Telemetry Systems, Isanti, MN, USA). We used the density of observations from winter aerial composition surveys to guide the spatial distribution of our sampling, attempting to deploy collars in the same proportion as observations. We collared 104 elk from the Avalanche Creek herd (2019–2021), 138 elk from the Bear's Ears and Steamboat herd (2019–2021), 113 elk from the Trinchera herd (2017–2021), and 158 elk from the Uncompahgre Plateau herd (2017–2021). See Supporting Information, Table A1 for year‐specific captures. All procedures were approved by the Colorado Parks and Wildlife Animal Care and Use Committee (protocol IDs: 02‐2017, 01‐2019, 02‐2019, 01‐2020, 03‐2020).

### 2.3 Overview of Analyses

To evaluate the role of study area, season, and movement strategy on resource selection, we classified individual elk‐years as either migrant or resident. Next, we estimated migratory departure and arrival dates to delineate ranges and seasonal space use of individuals within herds. We then extracted covariates from ranges and locations to generate seasonal, herd‐level resource use and selection models, which allowed us to compare responses among movement strategies. Lastly, we used a consistency index to characterize the overall degree of differentiation among herds, seasons, strategies, and groups of variables (Bastille‐Rousseau and Wittemyer 2022).

### 2.4 Movement Strategy Classification and Range Delineation

We used trends in Net‐Squared Displacement (NSD) and elevation shifts to classify movement strategies of individual elk. Following the methods of Bunnefeld et al. (2011), we classified elk as migrants or residents. We defined residents as individuals that occupied the same range year‐round or that commuted short distances, generally along an elevation gradient. We defined migrants as individuals moving seasonally between two distinct ranges. Additionally, we classified elk with consistent directional persistence as dispersers and elk with unclear movement strategies as ‘ambiguous’. We excluded individuals with insufficient data to confidently determine movement strategy (i.e., individuals with ≤ 5 months of data or other data issues such as collar malfunctions [*n* = 48]). We then delineated seasonal space use of individuals using the timing estimates of migratory movements. We produced initial parameter estimates by fitting a movement model using ‘MigrateR’ at the individual level, allowing us to estimate departure and arrival dates for migratory events (Spitz et al. 2017; see Supporting Information, Appendix A for more details).

We used a three‐range approach to delineate ranges. For most individuals (*n* = 154 migrants), we delineated ranges of their 1st winter, 1st summer, and 2nd winter. Some individuals were captured during or immediately before spring migration, resulting in an unclear or absent range in the 1st winter period. In these cases, we instead delineated ranges of their 1st summer, 2nd winter, and 2nd summer where data permitted (*n* = 70 migrants). To designate seasonal space use for residents, we took the median migration dates for each herd and subset seasonal periods from residents accordingly within that herd (Supporting Information, Appendix Table A3). We generated 95% contours of bivariate normal kernel density estimates (KDEs) with the reference bandwidth using the R package adehabitatHR to estimate seasonal ranges for each individual (Calenge 2019; Worton 1989). We included all collared elk with sufficient tracking data to estimate movement strategy frequencies (*n* = 465; *n* = 48 excluded due to data/tracking period issues). For resource selection analyses, we further excluded ambiguous (*n* = 5) or dispersing (*n* = 2) individuals and individuals with unclear seasonal space use (*n* = 72). We excluded these individuals due to difficulty in delineating seasonal home ranges and thus creating meaningful definitions of resource availability. Elk lying outside of the classical definitions of the migrant‐resident binary have been excluded from similar analyses for analogous reasons (van de Kerk et al. 2021).

### 2.5 Geospatial and Spatiotemporal Covariates

We selected suites of variables we hypothesized might reveal differences in resource selection among differing movement strategies. We grouped these variables into groups of covariates associated with specific resource types, representing measures of anthropogenic development, habitat, and vegetative productivity (Table 1). We scaled continuous variables before inclusion in models.

**TABLE 1:** Categories of covariates extracted for resource selection analyses.

Categories	Covariates for resource selection
Anthropogenic	Crops, development (distance to roads and residential landcover)
Habitat	Forest (reference category), herbaceous, other
Productivity	NDVI

Abbreviation: NDVI, normalized difference vegetation index.

We derived the Normalized Difference Vegetation Index (NDVI) from the MOD09Q1 Version 6 Moderate Resolution Imaging Spectroradiometer/Terra Reflectance product (MODIS; 250‐m spatial resolution, 8‐day temporal resolution; Vermote 2015). We calculated covariates representing pixel‐level inter‐annual and intra‐annual variation in NDVI metrics (predictability and seasonality respectively; see Appendix A for more details). We derived topography from the Shuttle Radar Topography Mission (SRTM, 30‐m resolution) dataset (Farr and Kobrick 2000). We extracted landcover covariates from the 2019 National Land Cover Database (30‐m resolution; Dewitz 2021) and consolidated them into five categories: residential, crop cover (typically irrigated grass or alfalfa fields), forest, herbaceous, and other (See Supporting Information, Appendix Table A2). We derived road‐related covariates from the TIGER county roads dataset (United States Census Bureau 2021). Distance layers were calculated for development (i.e., distance to residential landcover and roads). See Supporting Information, Appendix A for additional details regarding covariate acquisition and processing. An analysis linking landscape‐level patterns in environmental covariates and the prevalence of each strategy is presented in Supporting Information, Appendix B.

### 2.6 Third‐Order Resource Selection

We estimated resource selection functions (RSFs) for each strategy‐herd‐season combination using mixed‐effects exponential regression (Johnson et al. 2006). Using KDEs of seasonal ranges to define availability, we extracted all covariates (Table 1) from used locations and 10,000 randomly generated available locations per ID‐season‐year. We randomly assigned available locations to a specific date drawn with replacement from the range of dates in the corresponding season (Bastille‐Rousseau et al. 2015b). This framework allowed us to explore elk selection within seasonal ranges as a function of movement strategy. We generated resource selection functions using a binomial distribution, logit link, ID‐Season‐Year random intercepts, and random slopes for each variable (Muff et al. 2020). We excluded all variables with a Variance Inflation Factor ≥ 10 (VIF; Dormann et al. 2013; Graham 2003; Montgomery and Peck 1992). We natural log‐transformed distance variables and added 1 before inclusion in models to account for 0's. We conducted all analyses in program R version 4.2.2 (R Development Core Team 2022), using glmmTMB to fit mixed‐effects models (Brooks et al. 2017). We used forest as the reference category for landcover covariates. To further evaluate differences between migrants and residents, we also conducted an analysis comparing migrant and resident habitat use for each herd‐season (Bastille‐Rousseau et al. 2019; Latham et al. 2011). Full details and results are presented in Supporting Information, Appendix C.

### 2.7 Differentiation in Resource Selection

To understand which data strata (i.e., study area, season, movement strategies) most strongly structured resource selection behavior, we used a simple consistency score (Bastille‐Rousseau and Wittemyer 2022) calculated as the mean of the absolute difference between selection coefficients for herd *h* ∈ {Steamboat, Avalanche, Uncompahgre Plateau, and Trinchera} for a season level *s* ∈ {summer or winter} for a movement strategy *m* ∈ {migrant and resident} for a given covariate *k* ∈ {1…*n*}. We excluded Bear's Ears individuals from this analysis because they were all migrants. The consistency score C_hsmk_ can be aggregated across combinations of indices *h*, *s*, *m*, and *k* and is always positive, with values further from zero indicating greater differentiation (Wall et al. 2024).

We calculated and compared consistency across several combinations of indices *h* (herd), *s* (season), *m* (movement strategy), and *k* (covariate). We first combined all covariates *k* to evaluate which grouping had the highest differentiation. To do so, we calculated: (1) an average consistency score among herds (by comparing strategy‐season specific selection coefficients across each herd); (2) an average consistency score between seasons (by comparing herd‐strategy specific coefficients between seasons); and (3) an average consistency score between strategies (by comparing herd‐season specific coefficients between strategy). We also extracted season‐strategy consistency scores to evaluate if selection was more similar between strategies during the sympatric (winter) or allopatric period (summer). Additionally, we extracted herd‐specific consistency scores to evaluate if some herds had higher differentiation between migrants and residents in each season. We evaluated whether the prevalence of migrants in a population was linked to the consistency score by ordering them based on prevalence. Lastly, we re‐calculated these consistency scores by taking subsets of covariates *k* and grouping them into anthropogenic, habitat, and vegetation productivity covariates (Table 1). We carried forward uncertainty in the calculation of consistency scores by using Monte Carlo uncertainty propagation based on the standard errors of resource selection coefficients (Bastille‐Rousseau and Wittemyer 2022). We used Dunnett's T3 test to evaluate if any consistency scores differed statistically and ranked them (Dunnett 1980).

## 3 Results

### 3.1 Data and Movement Strategies

**FIGURE 2:**
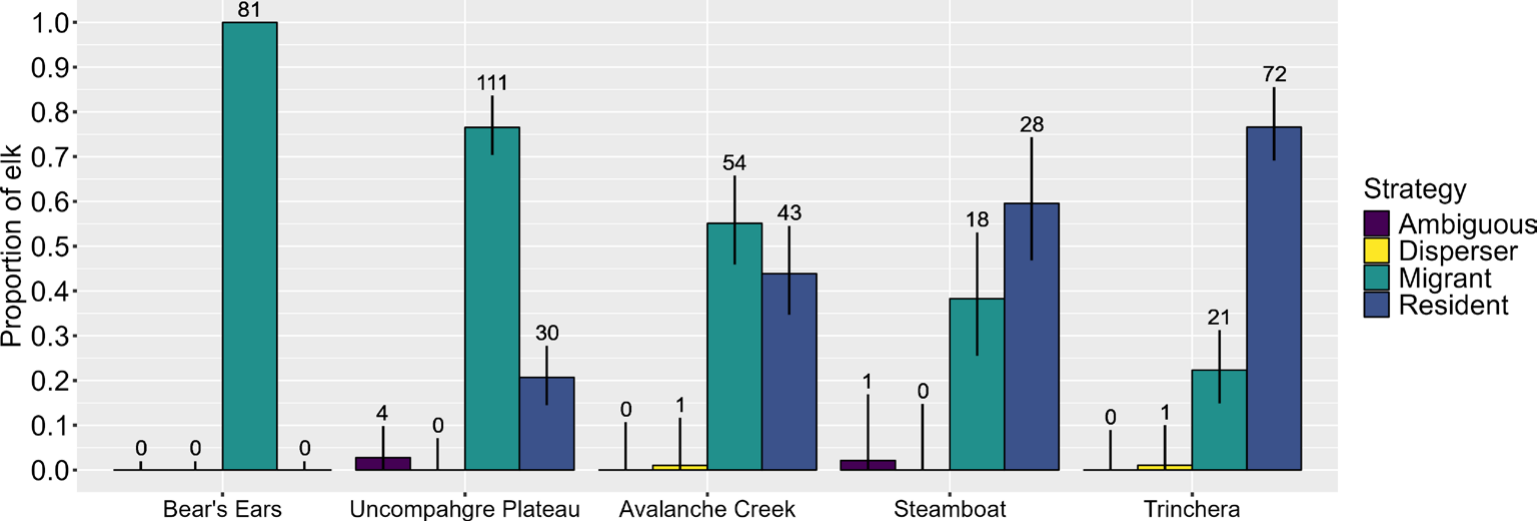
Proportion of movement tactics from 2017 to 2021 in the Avalanche Creek, Bear's Ears, Steamboat, Trinchera, and Uncompahgre Plateau elk herds in Colorado, USA. The number of elk‐years in each group is provided at the top of the 95% multinomial confidence intervals (black lines). Herds are ordered by decreasing estimated proportion of migrants.

We classified movement strategies for 465 elk‐years. We categorized 285 elk‐years as migratory, 173 elk‐years as resident, 5 elk‐years as ambiguous, and 2 elk‐years as dispersers, with movement strategy composition varying by study area and ranging from approximately 25%–100% migrants (Figure 2). We found intrapopulation and interpopulation variation in migratory movement parameters (e.g., date of departure, duration, distance; Supporting Information, Appendix D Tables D1 and D2). Qualitatively, landscape‐level patterns in each study area did not influence the prevalence of specific movement strategies (Supporting Information, Appendix B). We used 386 elk‐years in our further analyses (*n* migrant = 224, *n* resident = 162, *n* = 79 excluded), with a mean tracking duration of 342 days (± 53 days). On average, we used 4279 GPS locations per individual (± 1678 locations) with 1,651,836 GPS locations overall.

### 3.2 Resource Selection Models

**FIGURE 3:**
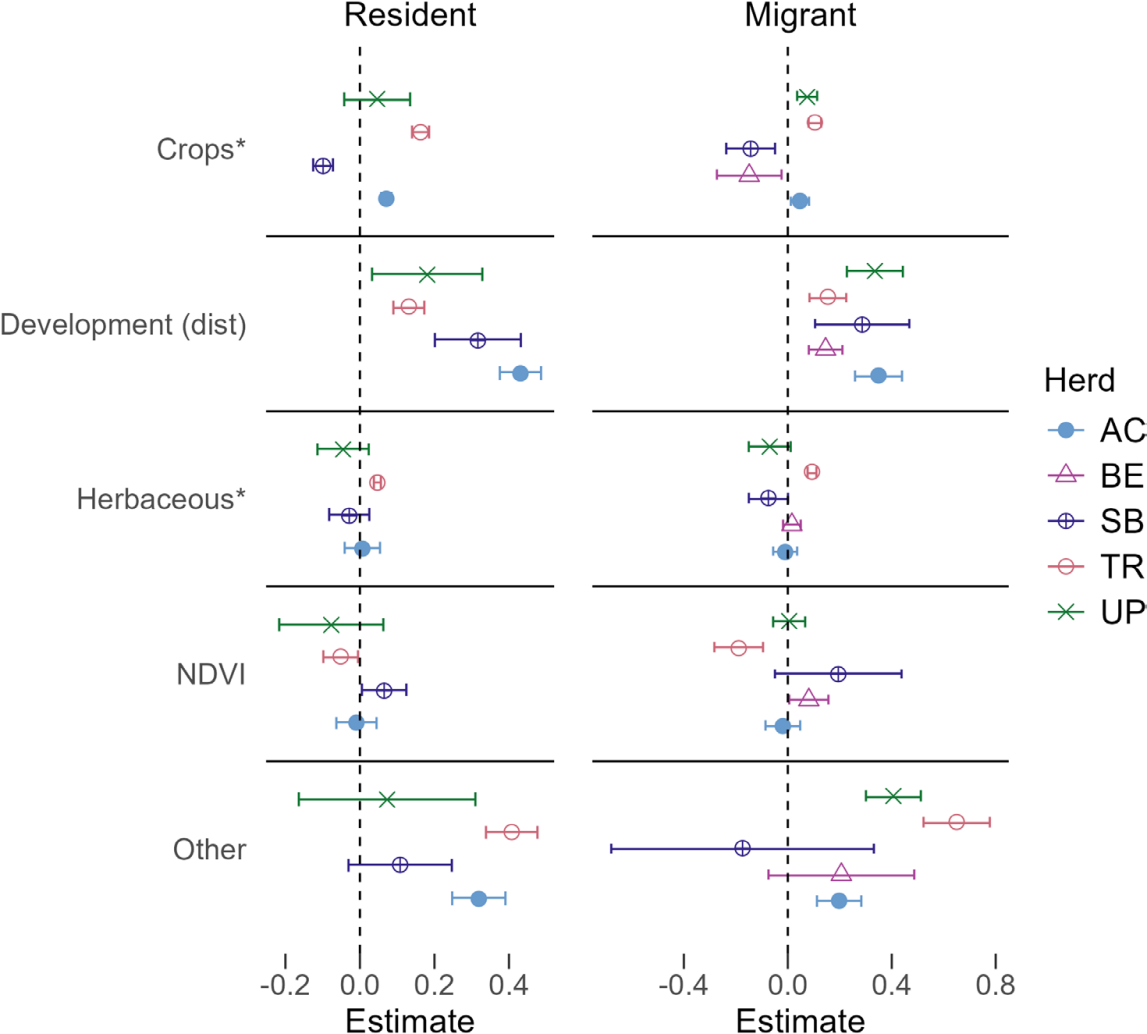
Estimates and 95% confidence intervals for each resource in strategy‐specific winter resource selection models. Confidence intervals above zero represent selection for a resource, while negative intervals represent avoidance. Covariates with an asterisk have been scaled for visualization purposes (see Table D2 for exact coefficient estimates). Herds are represented by their acronyms: AC, Avalanche Creek; BE, Bear's Ears; SB, Steamboat; TR, Trinchera; UP, Uncompahgre Plateau.

**FIGURE 4:**
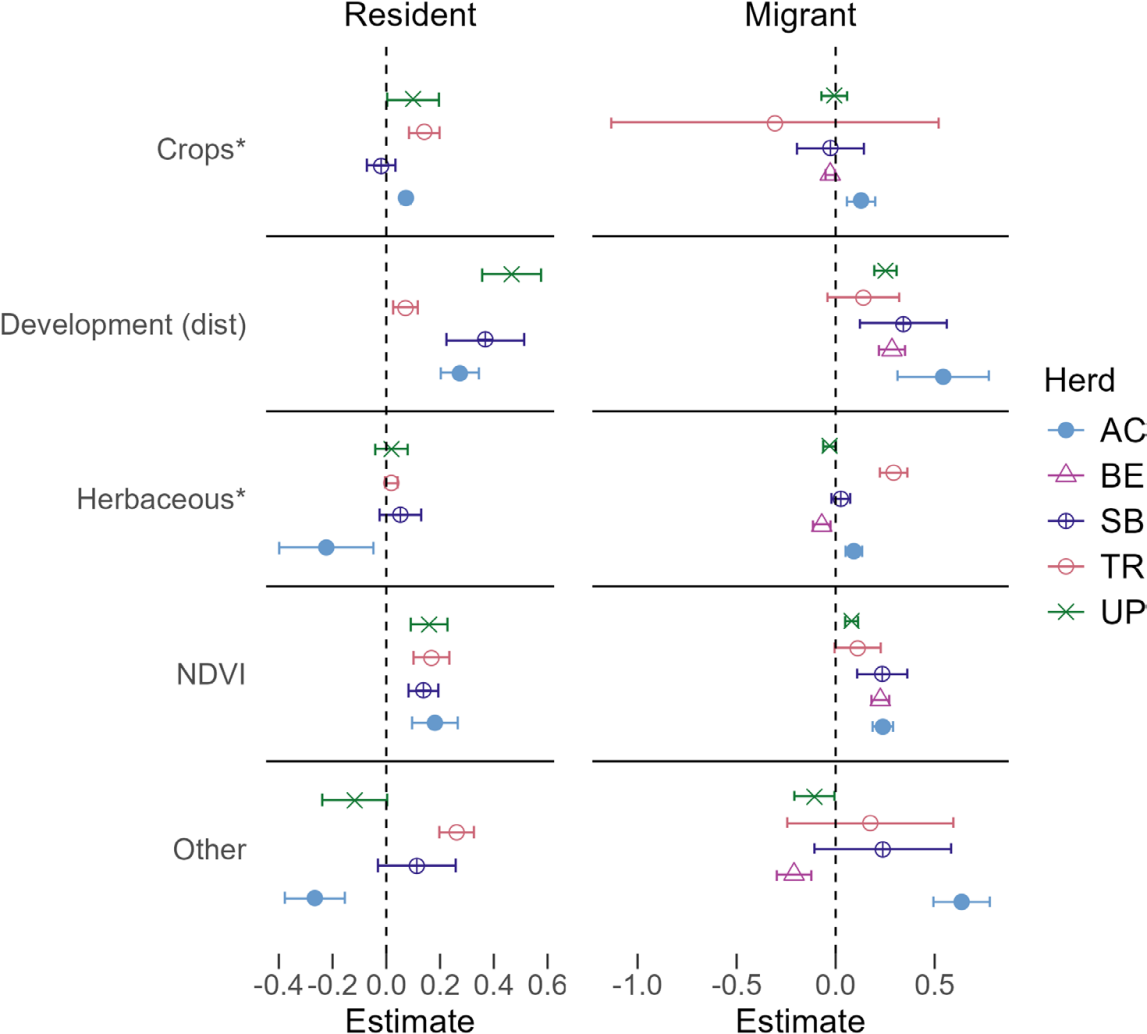
Estimates and 95% confidence intervals for each resource in strategy‐specific summer resource selection models. Confidence intervals above zero represent selection for a resource, while negative intervals represent avoidance. Covariates with an asterisk have been scaled for visualization purposes (see Table D4 for exact coefficient estimates). Herds are represented by their acronyms: AC, Avalanche Creek; BE, Bear's Ears; SB, Steamboat; TR, Trinchera; UP, Uncompahgre Plateau.

Patterns of resource selection in winter varied among herds and movement strategies (Figure 3). Summer resource selection models revealed response differences among movement strategies in multiple herds, with differing responses to herbaceous and other habitats in Avalanche Creek, herbaceous habitat in Trinchera, and distance to anthropogenic features in Uncompahgre Plateau (Figure 4). Similarly, our additional models revealed moderate differences in resource use between migrants and residents, primarily during the winter period, and inconsistent trends across herds and seasons (Supporting Information, Appendix C).

Strategy‐specific responses varied among herds and seasons, but some general trends were evident. Overall, elk selected areas farther from human development and roads (i.e., avoidance) regardless of strategy or season (17 positive estimates [+], 1 estimate no detected response [NR], i.e., confidence intervals overlapped zero). Response to crops (relative to forest) varied significantly by both herd and season. Winter models revealed a slightly higher likelihood for crop selection among elk (5+, 1 NR, 3 negative [−]), while summer models showed equal instances of positive selection and lack of response (4+, 4 NR, 1−). Response to habitat variables was also highly context‐dependent. In winter, selection of both habitat covariates was either positive or neutral compared to forest; responses to herbaceous habitat were largely neutral (2+, 7 NR), and other habitat responses were mixed between positive and lack of response (5+, 4 NR). By contrast, response to habitat covariates in summer was also mixed but additionally included avoidance compared to forest (herbaceous habitat: 2+, 5 NR, 2−; other: 2+, 4 NR, 3−). Lastly, while summer responses to NDVI were positive (8+, 1 NR), winter responses varied but skewed toward a lack of response (2+, 5 NR, 2−). Where strategy‐specific responses occurred within a herd, they were related primarily to habitat, though, as noted, differentiation in response to anthropogenic features was also detected in a few herds. We found no instances of strategy‐specific responses relating to vegetative productivity in either season.

### 3.3 Differentiation in Resource Selection

**FIGURE 5:**
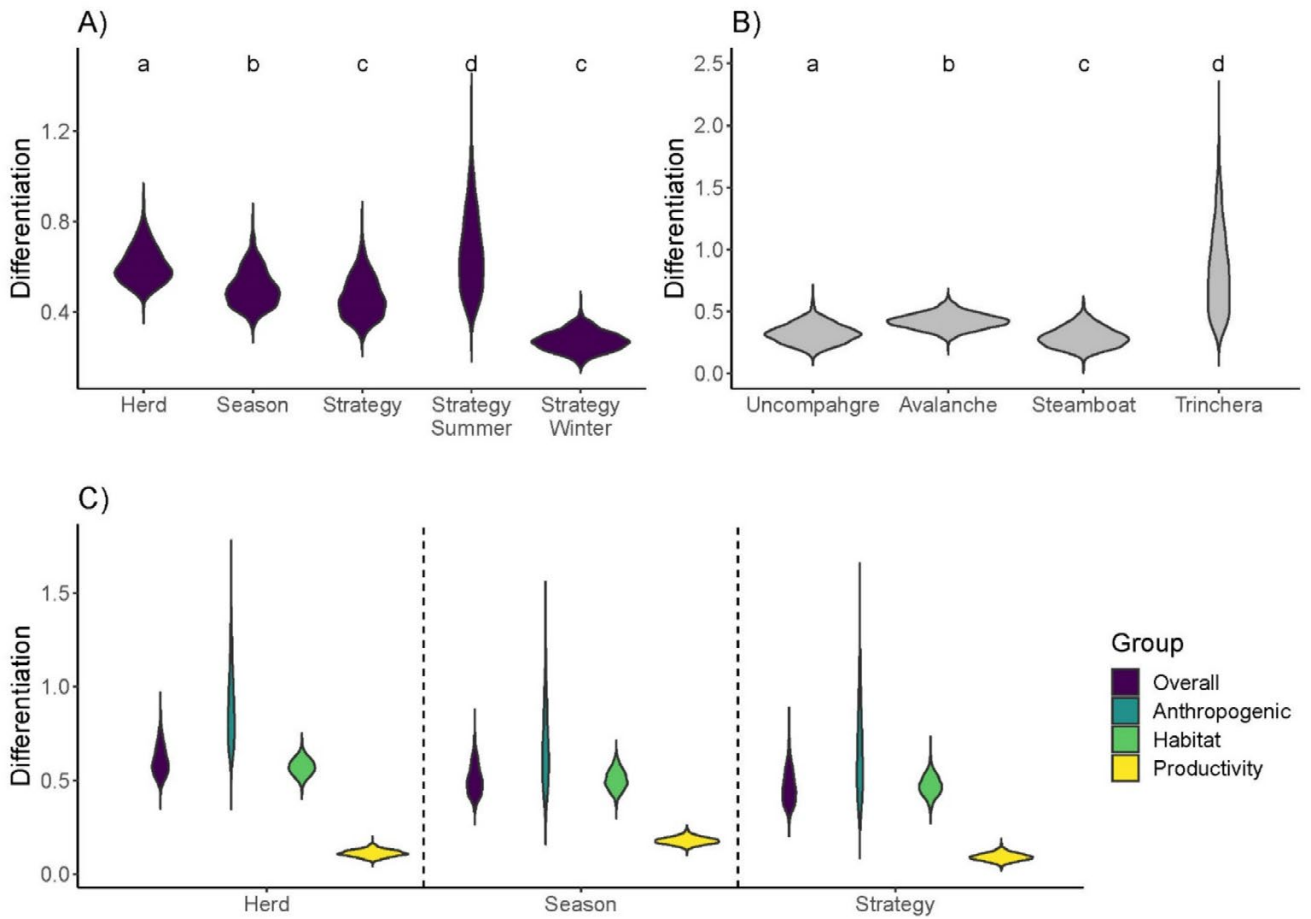
Violin plots of average consistency scores showing the degree of differentiation observed (A) between strategy, herd, season, and season‐strategy, (B) among individuals within each herd (ordered from most to least migratory), and (C) for different groupings (same as in panel A) in comparison to consistency scores calculated over a subset of covariates. For each grouping, all distributions were significantly different from the overall distribution. Consistency scores were calculated based on resource selection functions of 312 migratory and resident elk inhabiting four herds. Higher consistency scores indicate higher differentiation among individuals within that group. The distribution shown by each violin represents the distribution of the uncertainty around that score. Letters above each violin indicates significantly different distributions.

We generated several groupings of consistency scores indicating the degree of differentiation in elk behavior (Figure 5). Overall, elk behavior differed more by herd than by season and movement strategy (Figure 5A, *p* < 0.001 for all comparisons). Migrants and residents differed more during summer than in winter (Figure 5A, *p* < 0.001). Herds varied in how elk behavior changed over time (*p* < 0.001 for all comparison); however, the prevalence of each movement strategy did not seem to influence this relationship (Figure 5B, pearson *R* = −0.44, *p* = 0.554). For each grouping (strategy, season, herd), consistency scores were lowest for covariates associated with productivity (relative to the average score for that grouping), indicating that between strategies, seasons, or among herds, elk responses to these variables were relatively similar (Figure 5C). Elk behavior consistently differed more among the anthropogenic covariates, and this pattern was consistent across herds, seasons, and strategies.

## 4 Discussion

We evaluated movement strategy frequencies and differences in resource selection in four Rocky Mountain elk herds experiencing different environmental conditions. Overall, we found variation in the frequency of strategies within each herd and in selection behavior. This differentiation was herd‐specific, implying that strategy‐specific selection is complex and spatially and temporally contextual. Despite multiple herd‐specific differences, we also found some consistent similarities in selection. Elk responded negatively to human development at fine scales and selected areas with higher productivity during summer. Consistency scores highlighted that elk selection behavior differed most across herds, followed by variation between seasons and lastly by variation between strategies. Elk also responded more similarly to productivity and differed most in their responses to anthropogenic covariates. Overall, our work helps better understand the nuances in elk behavior across herds, seasons, and movement strategies while also providing information on the hierarchy of these factors in constraining elk resource selection.

### 4.1 Elk Movement Strategies and Resource Selection Behavior

We observed marked variation in the ratio of migrant and resident individuals across herds ranging from Bear's Ears being completely migrant to Trinchera being 77% resident (Figure 2). There was also variation among these herds regarding the timing, distance traveled, and duration of migration, indicating the potentially important role of spatial context on migratory frequency and parameters (Supporting Information, Appendix Tables D1 and D2). While environmental seasonality, variability, and predictability have been suggested as shaping the presence of movement strategies in different contexts (Bastille‐Rousseau et al. 2017; Mueller et al. 2011), these variables had a limited role in shaping strategy frequency across herds in this study (Supporting Information, Appendix B). Despite the low sample size, these patterns could indicate that the presence of different movement strategies might be driven by these large‐scale environmental patterns, but that the frequency of each strategy is driven by other processes such as density dependence and demographic success of each strategy as well as degree of plasticity in a species (Berg et al. 2019; Eggeman et al. 2016; Xu et al. 2021).

Our findings reinforce prior work demonstrating aversion to roads in elk and other ungulates (Montgomery et al. 2013; Passoni et al. 2021), and elk preference for areas with less human development (Buchanan et al. 2014; Morrison et al. 1995; Webb et al. 2011). Prior work has shown that crop cover can play an important but complex role in population migration frequencies and characteristics (Barker 2018; Barker et al. 2019; Jones et al. 2014). For example, the availability of irrigated agriculture on winter ranges of elk populations can discourage migration and increase frequencies of resident individuals, and delay migratory departure (Jones et al. 2014; Barker 2018), but this impact is reduced when high‐quality native forage is available (Barker et al. 2019). Our results reflect this nuanced response, as strategy‐specific responses to crop cover differed widely among study areas.

Elk selected strongly for NDVI in summer, with similar responses among strategies (Figure 4). This supports literature showing the importance of forage quantity in influencing ungulate movement in summer and suggests that responses are consistent among strategies (Lendrum et al. 2014; Mueller et al. 2011). We focused our analysis on vegetation quantity (NDVI) over vegetation quality (as measured through the instantaneous rate of growth; IRG) because preliminary results indicated NDVI was a better predictor of elk resource selection in our systems. Ungulates have been shown to select for vegetation quality along migratory corridors in some systems; however, (Aikens et al. 2017; Bischof et al. 2012; Merkle et al. 2016). A probable explanation for these findings is that elk are selecting for quantity over quality, as documented elsewhere (Hebblewhite et al. 2008; Merkle et al. 2016). Alternatively, IRG could be a poor measure of actual forage quality for elk.

### 4.2 Consistency in Resource Selection Behavior

In agreement with our predictions, we found more differentiation in elk selection behavior across herds than between seasons. The difference between herd and season was small, however (relative to the difference with strategy). We had expected there to be a stronger differentiation between herds and season than we observed, given the strong variation in terrain, climate, and habitat available to each herd. This indicates that temporal changes in resource availability experienced by each herd are similar to the consequences of spatial changes in the environment across herds in structuring elk behavior. Less surprisingly, differentiation between movement strategies within each herd and season was smaller, indicating that, despite using different movement strategies, within‐herd behavior remained similar. Also less surprisingly, elk behavior was most similar between strategies during the winter period when migrants and residents were more sympatric. This is likely explained by availability‐dependence in resource selection playing a large role in structuring resource selection behavior (Holbrook et al. 2019; Moreau et al. 2012). While each herd varied in how much behavior differed, these patterns were not related to the ratio of migrants and residents in a population. Interestingly, Trinchera, the population with the lowest proportion of migrants, also had the highest degree of differentiation. The Trinchera area is hotter and drier than our other study sites (U.S. Climate Data 2024), which may lead to more limiting resources and potentially more differentiation among the two movement strategies. A similar pattern has been observed elsewhere with African elephants (Bastille‐Rousseau and Wittemyer 2019).

When looking at differentiation as a function of subsets of covariates, we found that elk were most consistent in their selection for vegetation productivity. This is logical given the importance of foraging for any animal (Pettorelli et al. 2005). Differentiation was the highest for anthropogenic variables, despite all herds and strategies showing a relatively similar response to distance‐to‐anthropogenic features. This may be explained by the fact that crops were included in this category. As mentioned above, crops play a complex role in elk behavior, and responses may vary between strategies and herds (Barker 2018). While some studies have found strong differences between resident and migrant behavior in relation to development (Hebblewhite and Merrill 2009), this did not appear to be a driving force in our herds based on resource selection results for these covariates. Habitat‐related variables also had higher differentiation than productivity. Given the geographical extent of our study, part of the driving force behind this could be that specific land cover types (from a remote sensing classification) do not fulfill the same ecological function for elk, for example, switching from areas used to forage to areas used for cover. Creating site‐specific land cover classifications that integrate the functional role (e.g., forage or cover) would likely reduce the differentiation seen in this covariate category.

While initially developed as a way of comparing responses across individuals (Bastille‐Rousseau and Wittemyer 2022), consistency scores proved useful in comparing population‐level responses. In our application, calculating consistency scores across different groupings or strata (herd, season, strategy) allowed us to identify the hierarchy in the structuring factors of population‐level resource selection. Habitat‐selection functions are ubiquitous in movement ecology, yet synthesizing their inferences or comparing them across seasons or herds is non‐trivial. Summary metrics such as the consistency metric used here can assist in this task by focusing the inferences on the global responses of individuals or populations, not their specific responses to a set of covariates. We see value in comparing consistency across space and time to gather knowledge about spatiotemporal variation in the environment of different populations but also as a way of assessing the desired “level of inference” (i.e., herd‐season specific models vs. a single multi‐herd annual model). For example, if a system shows little seasonal differentiation but marked spatial variation, this will indicate that annual population‐specific inference should be of most interest. On the other hand, a system with strong seasonal differentiation and weak spatial differentiation would involve the need for season‐specific inferences but would allow one model that describes several populations.

Understanding the drivers of partial migration or other movement strategies within a system is a recurring topic in movement ecology. Using the consistency score (or other synthetic scores of resource selection) as a way of characterizing how allopatric or sympatric individuals display different movement strategies can help us better understand the link between strategies and resource selection. For example, a population with little differentiation between two movement strategies would be indicative of a system where resource selection itself is unlikely to cause the presence of different movement strategies. In a system where differentiation is mostly observed during the allopatric period (as observed here with elk), access to different resources during part of the year could be the cause or simply a consequence of the different strategies. A system where differentiation in resource selection is marked throughout the year would likely be indicative of a system where resource access is shaping movement strategies. Temporal changes in the frequency of migrants and residents are often evaluated concurrently with changes in each strategy's overall survival or recruitment (Middleton et al. 2013) or with changes in the broad environmental context (Williams et al. 2023). We see value in adding temporal change in behavioral consistency to these other factors.

Our results unveiled some of the complexity and variation in elk behavior. Overall, they emphasized not only that each herd differs in terms of habitat needs, but that these needs are at times strategy‐specific. Therefore, assessments of habitat suitability and protection of elk habitat need to take these different responses into account. Our results also highlighted a consistent negative response to anthropogenic disturbance, implying that continued development may negatively impact elk movement on seasonal ranges regardless of strategy.

## Author Contributions

**Storm Crews:** conceptualization (lead), data curation (lead), formal analysis (lead), investigation (equal), methodology (lead), validation (lead), visualization (lead), writing – original draft (lead), writing – review and editing (equal). **Nathaniel D. Rayl:** conceptualization (equal), data curation (equal), formal analysis (equal), funding acquisition (equal), investigation (equal), project administration (equal), writing – original draft (equal), writing – review and editing (equal). **Mathew W. Alldredge:** conceptualization (supporting), data curation (supporting), funding acquisition (supporting), investigation (supporting), project administration (supporting), writing – review and editing (supporting). **Eric J. Bergman:** conceptualization (supporting), data curation (supporting), funding acquisition (supporting), investigation (supporting), writing – review and editing (supporting). **Chuck R. Anderson Jr.:** conceptualization (supporting), funding acquisition (supporting), investigation (supporting), project administration (supporting), writing – review and editing (supporting). **Eric H. VanNatta:** conceptualization (supporting), data curation (supporting), funding acquisition (supporting), investigation (supporting), writing – review and editing (supporting). **Joseph D. Holbrook:** conceptualization (supporting), funding acquisition (supporting), investigation (supporting), writing – review and editing (supporting). **Guillaume Bastille‐Rousseau:** conceptualization (equal), data curation (equal), formal analysis (equal), funding acquisition (equal), investigation (equal), validation (equal), writing – original draft (equal), writing – review and editing (equal).

## Conflicts of Interest

The authors declare no conflicts of interest.

## Supporting information


Data S1.

## Data Availability

All data were collected by Colorado Parks and Wildlife. Data are available from the Dryad Digital Repository https://doi.org/10.5061/dryad.zkh1893mp.
