# Supplementary material for: Hierarchy in Structuring of Resource Selection: Understanding Elk Selection Across Space, Time, and Movement Strategies
Source: Ecol Evol. 2025 Mar 5;15(3):e71097. doi: 10.1002/ece3.71097 (PMC11882307; doi:10.1002/ece3.71097)
Supplement: Supplementary file 1 — Data S1. [file ECE3-15-e71097-s001.pdf]

APPENDIX A: ADDITIONAL METHODS

Movement Strategy Classification and Range Delineation

We followed the workflow of Spitz et al (2017) to classify movement strategies of individual elk-years. First, we fit a suite of net-squared displacement models to each individual elk-year, checked model fit to identify the movement characterization with the most support, and then extracted the migration parameter estimates from the top model. With this workflow, however, complex movement patterns may sometimes produce poor model fits, e.g., in the case of mixed-migrants (Cagnacci et al. 2016). When we had poorly fitting models, we visually assessed model fits and manually corrected misclassifications or poor parameter estimates, considering both displacement and elevation patterns. We then applied a corrective procedure to automated or manually derived estimates. We corrected departure date estimates by first generating a kernel density estimate (KDE) of space use using the temporal period immediately prior to the estimated departure date. We typically set this period to two weeks, but we altered it when necessary (e.g., when excursions or similar movements occurred close to the migration date, introducing spatial bias unrepresentative of the seasonal space use of an individual). We cropped locations during this period by the boundary of the 95% KDE and the last point within the KDE was assigned as the departure date estimate. We corrected arrival date estimates via an analogous process using the temporal period following the initial arrival date and identifying the first point in time within the KDE. We followed this procedure to improve accuracy and reduce bias in estimating seasonal range departure and arrival times. We resampled individual elk trajectories at the lowest available temporal resolution to avoid biased sampling among seasons (e.g., if an individual had a fix schedule that changed from 2-hour intervals in part of the year to 4-hour intervals in another part of the year, its trajectory was resampled from 12 points per day to 6 points per day).

We set a 30-day threshold to differentiate between migratory movements and excursion events, as these are superficially similar and the MigrateR workflow often struggles to differentiate them when

fitting movement models. If an individual departed and returned to a given seasonal range within 30 days, we considered this an excursion and not a migration (Cagnacci et al. 2016).

Geospatial and Spatiotemporal Covariates

To generate NDVI metrics, we downloaded raw MODIS imagery from 2007 to mid-2022 covering the full extent of the study area, reprojected to a local coordinate reference system. We followed the methodology of Bischof et al. (2012) and Merkle et al. (2016) to process imagery and fit a double-logistic curve to a time series of 8-day NDVI values on a per-pixel basis to interpolate daily NDVI estimates for each pixel. We developed covariates to measure the seasonality (intra-annual variation) and predictability (inter-annual variation) of NDVI. We calculated seasonality covariates on a per-pixel basis by annually calculating the standard deviation of the variable in question using daily interpolated values for each year from 2007-2021 and taking the mean of these annual values. We calculated predictability by extracting values on constant ordinal days across years and taking the standard deviation of these values per pixel (Bastille-Rousseau et al. 2017). For ease of interpretability, we transformed predictability covariates by subtracting values from 1 before modeling.

Our land cover categories included residential development, crop cover, forest, grassland/herbaceous, and other. Residential development included a combination of all intensities of residential cover available in the National Land Cover Database (NLCD) data. We derived road-related covariates from the TIGER county roads dataset, retaining primary, secondary, and local roads, paved areas, service roads, highway ramps and rural 4WD vehicular trails (Feature classes S1100, S1200, S1400, S1500, S1630, S1730, S1780 in dataset; US Census Bureau 2021). Distance covariates were calculated at a resolution of 30 meters.

Table A1. Number of elk collared in each herd.

**Herd**	**2017**	**2018**	**2019**	**2020**	**2021**	**Total**
Avalanche Creek	0	0	24	40	40	104
Bear’s Ears	0	0	2	70	66	138
Trinchera	23	21	30	19	20	113
Uncompahgre Plateau	23	25	30	40	40	158
Total	46	46	86	169	166	513

Table A2. Land cover categories used in analyses alongside the National Land Cover Database categories that comprise them.

**Land Cover Category**	**NLCD Category Number**	**NLCD Description**
Residential	21, 22, 23, 24	Developed (Open Space, Low, Medium, High)
Crop Cover	81, 82	Pasture/Hay, Cultivated Crops
Forest	41, 42, 43	Deciduous, Evergreen, Mixed Forest
Herbaceous	71	Grassland/Herbaceous
Other	-	-

Table A3. Herd-specific temporal delineation of seasonal periods as determined by migratory phenology. These values are in day of year (DOY) and were used to label resident locations as ‘summer’ or ‘winter’ by virtue of their timing.

Herd	End of Winter	Start of Summer	End of Summer	Start of Winter
Avalanche Creek	149	158	264	287
Steamboat	138	158	294	326
Trinchera	147	153	271	287
Uncompahgre Plateau	93	112	338	363

APPENDIX B: QUANTIFYING LANDSCAPE-LEVEL SPATIOTEMPORAL TRENDS IN FORAGE AND RELATIONSHIP TO FREQUENCY OF MOVEMENT STRATEGIES

Methods

We generated measures of spatiotemporal trends in forage for each study area, assessing averages in NDVI predictability and NDVI seasonality at the landscape scale. We also incorporated topography, generating averages and variability in elevation over each study area, given the a priori assumption that topographic heterogeneity likely affects forage at the scale of the study area. We generated 100% MCPs (Minimum Convex Polygons) for all available location data per herd and extracted these covariates within the MCPs. We then summarized them by taking the mean and standard deviation respectively. We fit linear models to investigate patterns between these metrics and the estimated percentage of migrants in each herd. We also fit simple LOESS models for visualization purposes, given that our sample size did not permit inclusion of quadratic (non-linear) effects.

Results

Landscape-level measures of predictability and seasonality of NDVI varied by study area (Appendix Figure B1). In general, we found no clear trends between landscape characteristics and percentage of migratory individuals within herds. Elevation trends were more predictive of migratory frequencies than forage predictability and seasonality metrics, but the relationship was very weak.

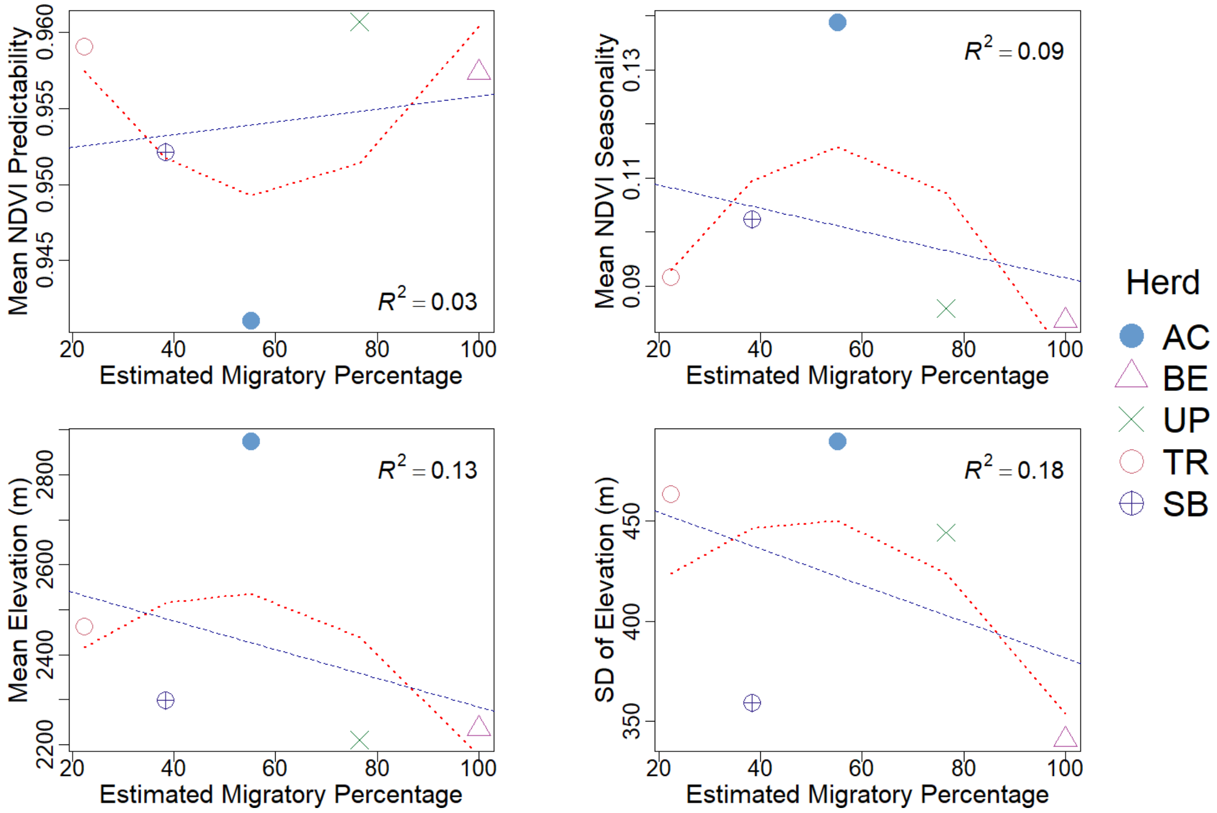

Figure B1. Metrics summarizing landscape characteristics in each study area and estimated percentage of migratory individuals. Metrics include averages in Normalized Difference Vegetation Index (NDVI) predictability, NDVI seasonality, and elevation over the full extent of each corresponding study area; additionally included is the standard deviation of elevation over the study area. Linear models fitted to the data are shown in blue, while LOESS curves fitted to the data are shown in red for visualization purposes. The R^2 value shown represents the goodness of fit for linear models. Herds are represented by their acronyms: AC = Avalanche Creek, BE = Bear's Ears, SB = Steamboat, TR = Trinchera, UP = Uncompahgre Plateau.

APPENDIX C: ASSESSING SEASONAL RESOURCE USE OF DIFFERING MOVEMENT STRATEGIES

We generated individual-level contours of 95% KDEs for every available seasonal period (e.g. 1st winter, summer, 2nd winter) to summarize seasonal range characteristics and examine differences in resource use between strategies (Table C1; Bastille-Rousseau et al. 2020). All covariates were summarized at the range level, with land cover covariates expressed as a percentage of total area and numeric covariates summarized as averages over space and time (where applicable). In contrast to the development variable used in our resource selection models, here development represents only the NLCD land cover values associated with residential development and does not incorporate roads.

We used logistic regression with movement strategy as a binomial response variable (migrant = 1, resident = 0). We generated separate models for each herd-season combination, including 1st and 2nd winter ranges, or 1st and 2nd summer ranges, within the same model. This resulted in summer and winter models for herds. We removed all variables with a Variance Inflation Factor ≥10 (VIF; Dormann et al. 2013, Graham 2003, Montgomery & Peck 1992). In one instance (Avalanche Creek summer model), a single covariate surpassed our VIF threshold for inclusion. For the sake of model structure conservation we ran two versions of this model: one with and one without the problematic covariate (NDVI). We presented the results for both versions of this model (Tables C1 and C2). We did not generate a model for the Bear's Ears herd because we classified all individuals in this herd as migrants.

Results

Use models revealed moderate differences in resource use for each strategy, especially during the winter period, and inconsistent trends across herds and seasons. Inclusion of the NDVI variable in our summer Avalanche Creek model altered parameter estimates for that herd, but did not impact results relating to strategy-specific use. During winter the ranges of migrants were more likely to be associated with higher average vegetative productivity compared to residents (2 instances of association with migrants [+], 2 instances of neutral association [NA]), while higher percentages of residential

development during this period were more likely to be associated with resident space use (2 instances of association with residents [-], 2 NA). Winter results for crop cover percentage suggested differences in use among strategies, but strategy association with higher values of this covariate varied among herds (2+, 1 NA, 1-). Summer models found fewer examples of strategy-specific resource use, only detecting differences in use of 'other' habitat with inconsistent trends (1+, 2 NA, 1-), and average vegetative productivity, which related to resident space use in one herd (1-, 3 NA).

Table C1. Categories of covariates extracted for resource use. Covariates were extracted and summarized at the seasonal range scale for use analysis. Acronyms: NDVI = normalized difference vegetation index

**Categories**	**Covariates for Resource Use**
Anthropogenic	Crops, Development
Habitat	Herbaceous, Other
Productivity	NDVI

Table C2. Table of winter use model coefficient estimates and associated 95% confidence intervals. Colors indicate the sign of a given estimate, with the direction of the coefficient representing whether a covariate was associated with migrant or resident space use. Green indicates association with migrant space use, red indicates association with resident space use, and yellow indicates that the confidence intervals of the estimate overlapped zero and thus we detected no strong association with either strategy.

Herd	Development	Crops	Herbaceous	Other	NDVI
Avalanche Creek	0.54 (0, 1.09)	-1.88 (-2.51, -1.25)	0.71 (-0.25, 1.67)	-0.96 (-1.49, -0.42)	0.63 (0.16, 1.11)
Steamboat	-0.73 (-1.61, 0.15)	-0.49 (-1.39, 0.42)	1.56 (-0.86, 3.98)	0.78 (-0.08, 1.64)	1.25 (0.2, 2.3)
Trinchera	-1.97 (-3.8, -0.14)	1.57 (0.49, 2.64)	0.01 (-0.8, 0.81)	-0.48 (-1.28, 0.31)	0.71 (-0.15, 1.56)
Uncompahgre Plateau	-1.71 (-3.01, -0.4)	2.56 (0.63, 4.49)	-0.16 (-0.66, 0.35)	-0.58 (-1.25, 0.09)	-0.17 (-0.94, 0.6)

Table C3. Table of summer use model coefficient estimates and associated 95% confidence intervals. Results for the Avalanche Creek summer model both inclusive and exclusive of NDVI are shown. Colors indicate the sign of a given estimate, with the direction of the coefficient representing whether a covariate was associated with migrant or resident space use. Green indicates association with migrant space use, red indicates association with resident space use, and yellow indicates that the confidence intervals of the estimate overlapped zero and thus we detected no strong association with either strategy.

Herd	Development	Crops	Herbaceous	Other	NDVI
Avalanche Creek (with NDVI)	-15.7 (-35.62, 4.22)	-5.61 (-13.94, 2.73)	5.68 (-5.57, 16.93)	-1.45 (-5.44, 2.53)	-3.17 (-9.07, 2.73)
Avalanche Creek (without NDVI)	-10.01 (-25.79, 5.78)	-4.13 (-11.62, 3.36)	10.61 (-3.65, 24.88)	-0.3 (-2.47, 1.86)	-
Steamboat	-0.42 (-2.86, 2.02)	0.13 (-0.68, 0.95)	1.18 (-0.41, 2.77)	1.78 (0.44, 3.13)	0.02 (-1.14, 1.17)
Trinchera	-0.93 (-2.43, 0.58)	0.71 (-0.17, 1.59)	-1.01 (-2.14, 0.12)	-3.11 (-4.8, -1.42)	-3.27 (-5.09, -1.46)
Uncompahgre Plateau	0.31 (-0.29, 0.91)	-0.08 (-0.67, 0.51)	-0.04 (-0.42, 0.34)	0.45 (-0.01, 0.9)	-0.03 (-0.48, 0.41)

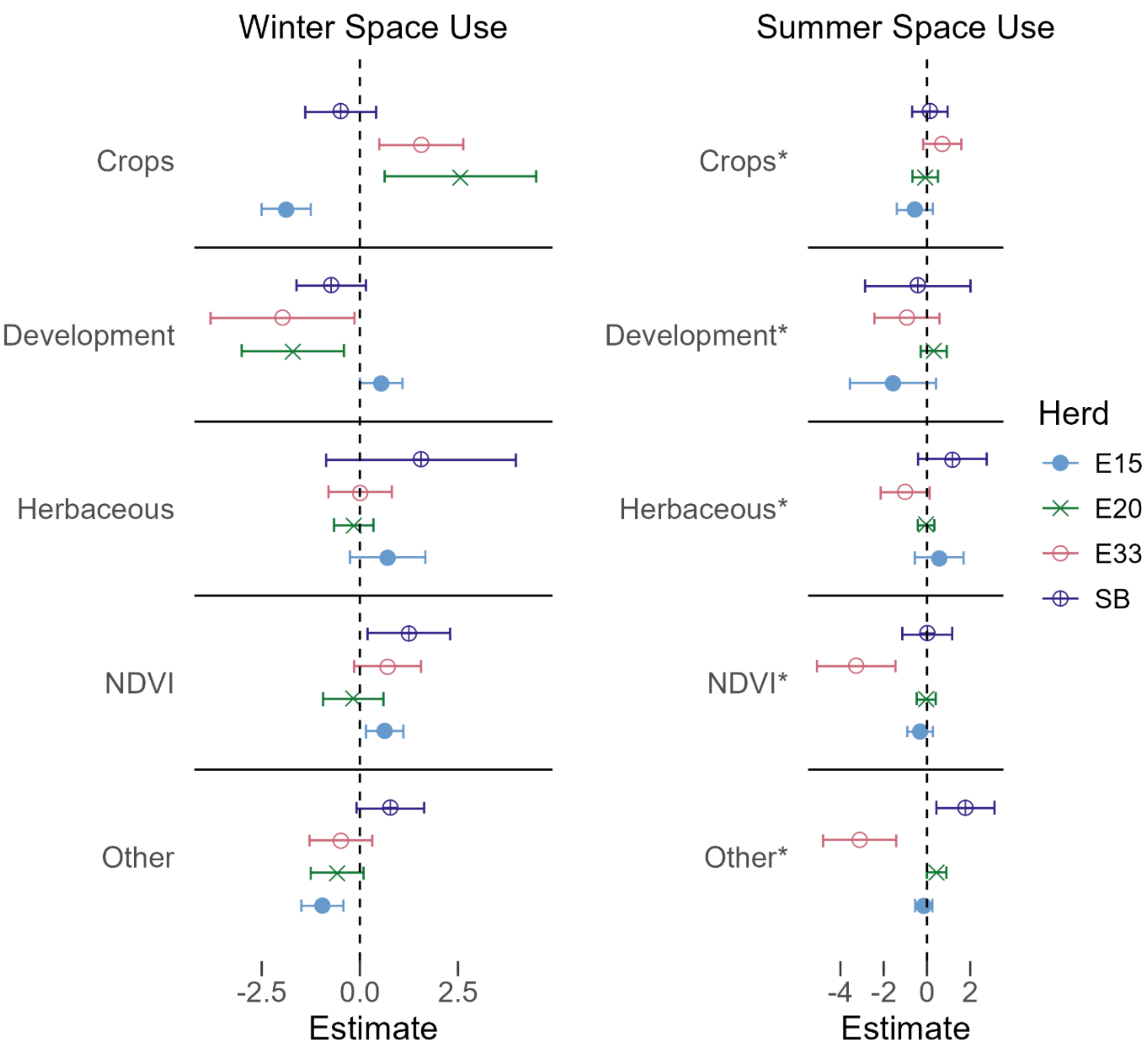

Figure C1. Estimates and 95% confidence intervals for each resource contrasting use in winter and summer between migrants and residents. Negative parameter estimates are associated with resident use while positive estimates are associated with migrant use. Note that summer estimates and confidence intervals for the Avalanche Creek herd were divided by ten for visualization purposes (marked with an asterisk). Refer to tables C1 and C2 for exact parameter estimates. Herds are represented by their acronyms: AC = Avalanche Creek, SB = Steamboat, TR = Trinchera, UP = Uncompahgre Plateau. Given that inclusion of NDVI in the summer Avalanche Creek model did not impact our findings, this figure plots results from the model inclusive of NDVI.

APPENDIX D: ADDITIONAL RESULTS

Table D1. Spring migration statistics for each herd. The initial value in sample size indicates the number of individuals with full migrations in their data set and from which migratory metrics were calculated. Parenthetical values in the Sample Size column indicate the number of individuals used to calculate departure dates, but where complete migrations were not available to calculate other metrics. Values in metric columns indicate the mean +/- the standard deviation, while numbers in brackets represent the minimum and maximum value respectively.

Herd	Sample Size	Departure Date (DOY)	Straight-line Distance (km)	Distance Traveled (km)	Duration (days)	Daily Displacement (km/day)	Elevation Difference (m)
Avalanche Creek	48 (49)	146.3 +/- 21.8 [99,220]	15.2 +/- 5 [6.6,24.9]	39.3 +/- 30.4 [6.8,138.8]	10.4 +/- 10.8 [0.25,43.6]	6.4 +/- 9.4 [0.4,40.1]	870 +/- 337 [0.26,1474]
Bear's Ears	72 (72)	94.6 +/- 12.1 [74,122]	80.3 +/- 21 [39.4,137.6]	191.9 +/- 79.9 [47.5,433]	30.1 +/- 17.9 [3.4, 86.3]	4.1 +/- 3.4 [0.9, 18.4]	337 +/- 210 [90,1114]
Uncompahgre Plateau	44 (59)	93.2 +/- 24.3 [40,180]	29.5 +/- 15.1 [5.2,87.9]	93.6 +/- 78.7 [8.6,374]	17.4 +/- 15.6 [0.7,67.5]	3.5 +/- 3.5 [0.5,15.2]	450 +/- 233 [64,1085]
Trinchera	17 (17)	148.3 +/- 12.9 [127,177]	14.3 +/- 5.2 [5.8,25.4]	37.5 +/- 26 [6.4,79.5]	8.9 +/- 8.4 [0.3,25.3]	4.6 +/- 5.4 [0.7,22.4]	768 +/- 384 [5,1254]

Steamboat	10 (12)	133.8 +/- 23.8 [90,181]	16.8 +/- 4.8 [10.1,23.9]	63.9 +/- 38.6 [15.1,120.6]	15.5 +/- 11.5 [1.3,30.9]	3.3 +/- 4.1 [0.5, 10.3]	581 +/- 318 [91,956]

Table D2. Fall migration statistics for each herd. The initial value in sample size indicates the number of individuals with full migrations in their data set and from which migratory metrics were calculated. Parenthetical values in the Sample Size column indicate the number of individuals used to calculate departure dates, but where complete migrations were not available to calculate other metrics. Values in metric columns indicate the mean +/- the standard deviation, while numbers in brackets represent the minimum and maximum value respectively.

Herd	Sample Size	Departure Date (DOY)	Straight-line Distance (km)	Distance Traveled (km)	Duration (days)	Daily Displacement (km/day)	Elevation Difference (m)
Avalanche Creek	42 (45)	272.9 +/- 34.6 [219,397]	13.8 +/- 4.6 [6.2,25.4]	93.8 +/- 73.4 [11.9,357.5]	32.8 +/- 27 [1.1,107]	2 +/- 3.8 [0.1, 17.2]	731 +/- 276 [3,1264]
Bear's Ears	50 (59)	319.5 +/- 32 [227,382]	81.3 +/- 23.8 [30.4,132.2]	268.9 +/- 145.3 [45.7,727,6]	47.5 +/- 32.3 [3, 133.4]	3.2 +/- 3.4 [0.6,17.8]	438 +/- 211 [46,971]
Uncompahgre Plateau	71 (71)	338 +/- 30.9 [250,447]	27.4 +/- 13.5 [5.9,87.4]	83.4 +/- 58.6 [8.4,239.2]	24.7 +/- 20.3 [0.9,78.3]	2.9 +/- 4.1 [0.3, 21.3]	533 +/- 252 [40,1115]
Trinchera	16 (17)	267.6 +/- 39 [209,335]	13.1 +/- 4.3 [5,22.5]	48.2 +/- 51.8 [5.7,187.3]	16.4 +/- 17.5 [0.8,62.8]	2.9 +/- 3.3 [0.3,11.2]	793 +/- 371 [53,1289]

Steamboat	12(12)	296.8 +/- 48.3 [208,369]	32.5 +/- 32.6 [9.1,105.3]	67.8 +/- 56 [15.5, 183.2]	14.4 +/- 13.1 [0.9,37.3]	5.5 +/- 5.5 [0.4,15.8]	333 +/- 179 [37,771]

Table D3. Table of winter resource selection function coefficient estimates and associated 95% confidence intervals. Colors represent the sign of a given estimate, with green being a positive estimate, red being a negative estimate, and yellow being a neutral estimate (i.e. the confidence intervals overlap zero and we cannot be confident that the response is positive or negative). An asterisk denotes a difference in response between strategies within a herd.

Herd	Distance to Development	Crops	Herbaceous habitat	Other habitat	NDVI
Avalanche Creek Migrants	0.35 (0.26, 0.44)	0.47 (0.12, 0.82)	-0.10 (-0.56, 0.36)	0.20 (0.11, 0.28)	-0.02 (-0.09, 0.05)
Avalanche Creek Residents	0.43 (0.38, 0.49)	0.71 (0.57, 0.85)	0.06 (-0.41, 0.54)	0.32 (0.25, 0.39)	-0.01 (-0.06, 0.04)
Bear's Ears Migrants	0.15 (0.08, 0.21)	-1.49 (-2.73, -0.24)	0.16 (-0.18, 0.5)	0.21 (-0.07, 0.49)	0.08 (0.01, 0.16)
Steamboat Migrants	0.29 (0.1, 0.47)	-1.43 (-2.37, -0.49)	-0.75 (-1.50, 0.01)	-0.17 (-0.68, 0.33)	0.19 (-0.05, 0.44)
Steamboat Residents	0.32 (0.2, 0.43)	-0.99 (-1.25, -0.72)	-0.29 (-0.83, 0.25)	0.11 (-0.03, 0.25)	0.06 (0.01, 0.12)
Trinchera Migrants	0.15 (0.08, 0.22)	1.04 (0.8, 1.28)*	0.93 (0.76, 1.10)*	0.65 (0.52, 0.78)*	-0.19 (-0.28, -0.10)
Trinchera Residents	0.13 (0.09, 0.17)	1.63 (1.4, 1.86)*	0.47 (0.37, 0.56)*	0.41 (0.34, 0.48)*	-0.05 (-0.10, -0.01)

Uncompahgre Plateau Migrants	0.34 (0.23, 0.44)	0.74 (0.36, 1.13)	-0.70 (-1.50, 0.11)	0.41 (0.30, 0.51)	0.01 (-0.06, 0.07)
Uncompahgre Plateau Residents	0.18 (0.03, 0.33)	0.46 (-0.42, 1.35)	-0.45 (-1.14, 0.24)	0.07 (-0.16, 0.31)	-0.08 (-0.22, 0.06)

Table D4. Table of summer resource selection function coefficient estimates and associated 95% confidence intervals. Colors represent the sign of a given estimate, with green being a positive estimate, red being a negative estimate, and yellow being a neutral estimate (i.e. the confidence intervals overlap zero and we cannot be confident that the response is positive or negative). An asterisk denotes a difference in response between strategies within a herd.

Herd	Distance to Development	Crops	Herbaceous habitat	Other habitat	NDVI
Avalanche Creek Migrants	0.54 (0.31, 0.77)	1.29 (0.57, 2.00)	0.46 (0.25, 0.67)*	0.64 (0.49, 0.78)*	0.24 (0.19, 0.29)
Avalanche Creek Residents	0.27 (0.20, 0.34)	0.73 (0.55, 0.91)	-1.12 (-1.99, -0.24)*	-0.27 (-0.38, -0.15)*	0.18 (0.10, 0.27)
Bear's Ears Migrants	0.28 (0.22, 0.35)	-0.27 (-0.51, -0.03)	-0.35 (-0.57, -0.13)	-0.21 (-0.3, -0.12)	0.23 (0.18, 0.27)
Steamboat Migrants	0.34 (0.12, 0.56)	-0.26 (-1.95, 1.43)	0.13 (-0.11, 0.37)	0.24 (-0.11, 0.58)	0.24 (0.11, 0.36)
Steamboat Residents	0.37 (0.22, 0.51)	0.19 (-0.72, 0.34)	0.26 (-0.13, 0.65)	0.11 (-0.03, 0.26)	0.14 (0.08, 0.19)
Trinchera Migrants	0.14 (-0.04, 0.32)	-3.06 (-11.33, 5.20)	1.46 (1.12, 1.81)*	0.18 (-0.24, 0.59)	0.11 (-0.01, 0.23)
Trinchera Residents	0.07 (0.03, 0.12)	1.41 (0.84, 1.98)	0.09 (-0.02, 0.21)*	0.26 (0.20, 0.33)	0.17 (0.10, 0.23)

Uncompahgre Plateau Migrants	0.25 (0.20, 0.31)*	-0.07 (-0.72, 0.58)	-0.15 (-0.31, 0.01)	-0.11 (-0.21, -0.01)	0.08 (0.05, 0.11)
Uncompahgre Plateau Residents	0.47 (0.36, 0.58)*	1.00 (0.03, 1.96)	0.10 (-0.21, 0.40)	-0.12 (-0.24, 0.00)	0.16 (0.09, 0.23)
